# Chopped Radiation Measurements With Large Area Si Photodiodes

**DOI:** 10.6028/jres.103.009

**Published:** 1998-04-01

**Authors:** George Eppeldauer

**Affiliations:** National Institute of Standards and Technology, Gaithersburg, MD 20899-0001

**Keywords:** Bode plot, chopped light, detector, frequency dependence, gain, measurement, optical radiation, photocurrent, photodiode, radiometer, responsivity, silicon

## Abstract

Frequency dependent response characteristics of photocurrent meters using large area, radiometric quality Si photodiodes have been analyzed. The current responsivity, the voltage noise and drift amplification, and the gain and bandwidth of the photocurrent-measuring analog control loop were calculated. The photodiodes were selected for high shunt resistance. The effect of the photodiode junction capacitance on the response characteristics was also analyzed. As a result of photocurrent gain dependent frequency compensations, the noise boosting effect was minimized at the output of the current meter. The loop gain and bandwidth were maximized. High-accuracy photocurrent measurements can be achieved using the described procedures for both dc and modulated optical radiation.

## 1. Introduction

In many applications, the signal of optical radiation varies rapidly. Also, the optical radiation can be modulated or chopped. In these applications, the frequency dependent signal and noise gains of a photocurrent meter are to be optimized. In order to maximize the performance of the current meters for different photodiodes, their frequency dependent current-to-voltage (transimpedance) and voltage gains will be determined and the control loop gain and bandwidth will be optimized for the signal frequencies at all signal-gain ranges.

The dc and low-frequency characteristics of low-photocurrent measuring circuits of large area silicon photo-diodes were analyzed earlier [1,2,3]. It was shown that for high sensitivity photocurrent measurements, high feedback resistors and high shunt resistance photodiodes are required. The high source resistance (parallel combination of the feedback and shunt resistances) requires current measuring operational amplifiers with low input bias currents. Such devices are the ultra low bias current Field Effect Transistor (FET) or the dielectrically isolated FET operational amplifiers. The high source resistance produces high thermal (Johnson) voltage noise, and the FET input stage of the operational amplifier has a large 1/*f* voltage noise. These dominant voltage noise sources were equalized when a measurement time of 400 s was used. Drifts and noise were equalized by regulating the device temperature [4] with an instability smaller than +/− 0.02 °C. The photodiodes were selected for a shunt resistance close to 10 GΩ in order to keep the voltage amplification close to unity. The equivalent photocurrent of the equalized noise and drift was 0.1 fA with an electrical bandwidth of 1.25 mHz. This implies a dynamic range of 14 orders of magnitude (10^14^) for a silicon photodiode current meter. The time constants (shunt resistance times junction capacitance) of the tested silicon photodiodes varied between 10 s and 200 s. When the bandwidth was increased to 300 mHz, the output noise of the photocurrent-measuring circuit increased significantly. The sensitivity decrease for the large-time-constant photodiodes was about a factor of 10. This was an indication of a frequency dependent noise boosting effect in the noise amplification of the current meter.

The optical radiation first is converted into an electrical signal by the photodiode itself; then, the electrical signal of the photodiode is measured. In radiometric applications, current measuring preamplifiers are connected to the photodiode. The internal speed of the photodiode depends on the time needed to convert the accumulated charge into current. The photodiode internal conversion time is determined by the diffusion time of carriers generated outside the depletion layer and the carrier transit time in the depletion layer [5]. The maximum frequency at which modern silicon photodiodes will produce current is somewhere in the 2 GHz range [6], depending on the area of the detector and the type of silicon material used. It is important to keep the internal capacitance of the photodiode low because this capacitance will produce an additional time constant in conjunction with the load resistance. This time constant *τ* works like a low-pass filter for the current of the photodiode. The junction capacitance is proportional to the active area and inversely proportional to the square or cube root of the width of the depletion layer. The depletion layer width is proportional to the product of the resistivity of the material and the reverse voltage [5] (if it is applied to the diode). The silicon bulk resistivity can be specified from 1 Ω cm to 10000 Ω cm [6]. The higher the resistivity, the lower the junction capacitance of the photodiode.

Most frequently, the photodiode rise time is reported instead of its 3 dB response roll-off frequency. The rise time is the time required for the output signal of the photodiode to change from 10 % to 90 %. For different types of large area silicon photodiodes the rise time is between 1 μs and 7 μs when a 1 kΩ load resistor and zero biasing voltage are applied [5]. An average junction capacitance for large area silicon photodiodes is about 1 nF. This gives a *τ* = 1 μs time constant with the 1 kΩ load resistor at test. This is equivalent to a 3 dB signal response roll-off frequency of 160 kHz. Caution is necessary when the modulation frequency of the measured optical radiation is close to or higher than the 3 dB frequency. At those high frequencies the modulated optical signal can be damped because of the amplitude roll-off, resulting in a changing ac photocurrent response versus frequency. In these cases, ac to dc photocurrent conversions, realized by low pass filters, give higher accuracy than ac signal measurements. This idea was successfully utilized in mode-locked laser measurements using two different kinds of large area silicon photodiodes [7]. The laser pulse width was 5 ps, the repetition rate was 100 MHz, and the averaged (dc) photocurrent was measured. The responses of the photodiodes were equal for both pulsed and cw laser measurements.

The type of the load resistor or preamplifier connected to the terminals of the photodiode plays an important role in the response time of the overall optical radiation measurement. If the photodiode current is converted into a voltage through a single load resistor the dominating time constant will be the product of the photodiode capacitance and the load resistance. In order to measure the short circuit current, which is proportional to the detected optical power, the load resistor has to be much smaller than the photodiode resistance. Instead of a small load resistor, a current-to-voltage converting analog control loop can be used as a preamplifier [8]. A current meter like this has a low input impedance and a high current-to-voltage gain. However, the magnitude of the complex input impedance varies with frequency. This impedance is connected in parallel with the photodiode junction capacitance. The transformation of the feedback impedance to the input of the current meter and the frequency dependent diode current-to-voltage response will be determined below.

The advantage of ac signal measurements is that the measuring circuit can be made selective for the signal frequency. This way a narrow measurement bandwidth can be chosen close to the elbow of the preamplifier’s 1/*f* noise range where the 1/*f* noise is small.

The shunt resistance and the junction capacitance of different large area Si photodiodes can change significantly. It is important to understand the effect of the photodiode impedance on the optical radiation measuring analog control loop when high sensitivity and fast operations are expected. The analog control loop will produce a high accuracy current-to-voltage conversion only if the loop gain and bandwidth are high enough at the signal frequencies. Also, the current measuring analog control loop, coupled to the output of the photodiode, is the first stage of the measuring circuit. The first stage dominates the signal-to-noise ratio for the output of the measuring circuit. It is important to keep the first stage amplification for the dominant voltage noise low within the electrical bandwidth of the measurement. The electronic circuits in the second stage, such as a lock-in amplifier or a digital voltmeter, cannot restore the established signal-to-noise ratio caused by the lack of optimization in the first stage.

In the present work, the previously described dc and low frequency analysis of large area and wide dynamic range Si photodiode current meters is extended to higher signal frequencies. The purpose of this paper is to show how to maximize the performance of photocurrent meters for different photodiodes at different signal frequencies.

## 2. Photodiode Current Measuring Circuit

**Fig. 1:**
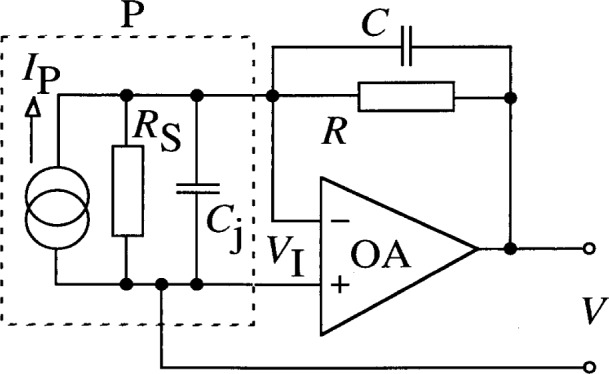
Simplified circuit diagram of a photocurrent meter. The dashed line represents the photodiode P, and R and C are the feedback components of the operational amplifier OA.

In order to achieve linear operation in a wide dynamic signal range, the short-circuit current of the photodiode has to be measured. The simplified scheme of widely used short-circuit current meters is shown in Fig. 1.

The photocurrent *I*_P_ of the photodiode P is measured by a current-to-voltage converter. The current-to-voltage conversion is realized by an operational amplifier OA. P has a shunt resistance *R*_S_ and a junction capacitance *C*_j_ which together produce the photodiode impedance *Z*_d_. *I*_P_ is converted into a voltage *V* through the feedback impedance of the OA. The feedback impedance *Z* is the parallel connection of the feedback resistor *R* and its parallel capacitance *C*. The OA input voltage *V*_I_ is small because of the large OA open loop gain, *A*. As the maximum of *V* is 10 V, and *A* is about 10^6^, *V*_I_ is equal or smaller than 10 μV. This very small voltage drop on P produces a small load resistance *R*_I_ for the photodiode^3^:

$$R_{I}\approx \frac{R}{A}.$$

Determination of *R*_I_ is only sufficient for dc or low frequencies. It is the input impedance *Z*_I_ of the current meter that determines the time constant *τ* of the photodiode short-circuit current measurement at higher frequencies:

$$Z_{I}=\frac{Z}{A}=\frac{R}{A}\frac{1}{\left(1+j\omega \;CR\right)}=\frac{1}{\frac{A}{R}+j\omega \;AC}.$$ (1)

The rewritten (popular) form of Eq. (1) shows that the input impedance of the current meter can be calculated as the parallel connection of the down-transformed feedback resistor and the up-transformed feedback capacitor of the OA:

$${\left(Z_{I}\right)}^{-1}={\left(\frac{R}{A}\right)}^{-1}+{\left(\frac{1}{j\omega \;AC}\right)}^{-1}.$$

*C*_j_ will be increased significantly by the parallel connected, up-transformed feedback capacitor *AC*. If *R*/*A* << *R*_S_, the measurement time constant is

$$\tau =\frac{(C_{j}+AC)R}{A}.$$ (2)

As an example, *AC* = 2 μF if *C* = 2 pF and *A* = 10^6^. For *R* = 10^9^ Ω, the input resistance will be *R*_I_ = 10^3^Ω. AC >> *C*_j_ for *C*_j_ = 1 nF; therefore, *τ* = *RC* = 2 ms. *Z* and the impedance of the photodiode *Z*_d_ create a feedback network from the OA output to the OA input. The voltage attenuation of the feedback network is *β*.

For accurate dc and/or ac photocurrent measurements, the photodiodes have to be selected for shunt resistance and junction capacitance; and the other passive components of the feedback network are to be calculated. Thereafter, an OA has to be selected which satisfies the noise, drift, input current, and speed (bandwidth) requirements.

### 2.1 Drift and Noise Amplification

The OA input voltage-noise, which is the principal source of 1/*f* noise, and input offset-voltage with its drift [1,2,3], are amplified to the OA output by the closed loop voltage gain *A_V_* of the photocurrent measuring analog control loop [9,10]:

$$A_{V}=\frac{A}{1+G}=\frac{1}{\beta }\frac{1}{1+G^{-1}},$$ (3)

where the loop gain is *G* = *Aβ*. According to Eq. (3), if *G* >> 1, the feedback network determines *A_V_*. The feedback attenuation is:

$$\beta \approx \frac{1}{A_{V}}=\frac{Z_{d}}{Z_{d}+Z},$$ (4)

where (*Z*_d_)^−1^ = (*R*_S_)^−1^ + *jω C*_j_ and *Z*^−1^ = *R*^−1^ + *jω C*.

Therefore,

$$\begin{array}{l} \beta =\frac{R_{s}(1+j\omega RC)}{R_{s}(1+j\omega RC)+R(1+j\omega R_{s}C_{j})}\\ \;=\frac{R_{s}(1+j\omega RC)}{R_{s}+R+j\omega (RR_{s}C+RR_{s}C_{j})}. \end{array}$$

With

$$\beta_{o}=\frac{R_{s}}{R_{s}+R},$$

the feedback attenuation can be written as:

$$\beta =\beta_{o}\frac{1+j\omega RC}{1+j\omega \frac{RR_{s}C+RR_{s}C_{j}}{R+R_{s}}}.$$ (5)

The dc (or very low frequency) voltage amplification of the photocurrent measuring circuit is the reciprocal of *β*_o_:

$$A_{Vo}=\frac{R_{s}+R}{R_{s}}=1+\frac{R}{R_{s}}.$$ (6)

The frequency dependent closed loop voltage gain can be written as the reciprocal of *β* in Eq. (5):

$$A_{V}=\frac{R_{s}+R}{R_{s}}\frac{1+j\omega \frac{RR_{s}C+RR_{s}C_{j}}{R+R_{s}}}{1+j\omega RC},$$ (7)

where the integrating time constant is *τ*_1_ = *RC*. The differentiating time constant is

$$\tau_{2}=\frac{RR_{s}C+RR_{s}C_{j}}{R+R_{s}}=\frac{RR_{s}}{R+R_{s}}(C+C_{j}).$$ (8)

*τ*_2_ is calculated from the product of two factors. One factor is the resultant resistance of the parallel connected *R* and *R*_S_. The other factor is the sum of *C* and *C*_j_ [10].

For fast operation, *C* has to be small. However, *C* has a minimum value of 1 pF to 2 pF, because of the stray capacitance parallel to *R*. For all large area photodiodes, *C*_j_ >> *C* when the external *C* is not connected parallel to *R*. In this case, the frequency dependent voltage amplification of the measuring circuit can be written as:

$$A_{V}=A_{Vo}\frac{1+j\omega \frac{RR_{s}C_{j}}{R+R_{s}}}{1+j\omega RC}.$$ (9)

If *R* >> *R*_S_ the voltage amplification is:

$$A_{V^{\left(1\right)}}=A_{Vo}\frac{1+j\omega R_{s}C_{j}}{1+j\omega RC}.$$ (10)

In this case, the differentiating time constant in the numerator is determined exclusively by the impedance of the photodiode. If *R*_S_ >> *R*, then

$$A_{V^{\left(2\right)}}=A_{Vo}\frac{1+j\omega RC_{j}}{1+j\omega RC}.$$ (11)

In Eq. (3),

$$A=A_{o}\frac{1}{1+j\omega \tau_{i}},$$ (12)

where *τ*_i_ is the integrating time constant of the OA. *A*_o_ is the dc open loop gain of the OA.

### 2.2 Frequency Dependent Signal Response

The frequency dependent current-to-voltage response of the photocurrent measuring circuit can be calculated as well. With the Norton to Thevenin source conversion formula [11] the photocurrent *I*_P_ can be converted into *V*_I_:

$$V_{I}=I_{P}\frac{1}{1+j\omega R_{s}C_{j}}.$$ (13)

As a next step, *V*_I_ is amplified by the OA. The voltage amplification here [9] is equal to the ratio of *Z* to *Z*_d_:

$$\frac{V}{V_{I}}=\frac{R}{R_{s}}\frac{1+j\omega R_{s}C_{j}}{1+j\omega RC}.$$ (14)

When Eq. (13) is substituted into Eq. (14) the photocurrent-to-voltage conversion [13,14] *A_I_* which is frequently called the transimpedance gain, can be written as:

$$A_{I}=\frac{V}{I_{P}}=R\frac{1}{1+j\omega RC}.$$ (15)

Equation (15) shows that the dc signal gain (or response), which is the ratio of the output voltage *V* to the input photocurrent *I*_P_ is equal to *R*. The frequency dependent signal response is determined by the integrating time constant, *τ*_1_ = *RC*, of the feedback impedance. This time constant and the OA input-impedance-determined time constant *τ*, in Eq. (2), are the same: *τ*=*τ*_1_ = *RC*. Equation (15) also describes the gain for the input current noise of the circuit. Because Eq. (15) does not show the frequency dependent contribution of the OA to the signal response, the equation works only if *τ*_i_ < *τ*_1_. Otherwise, the OA frequency dependent response can give an unwanted limitation for the signal response. Similarly to Eq. (3),

$$\frac{V}{I_{P}}=R\frac{1}{1+G^{-1}}.$$ (16)

The current-to-voltage conversion, *R*, which is the signal gain, will be accurate only if *G* >> 1 at the signal frequency. This is a very important design requirement for the analog control loop.

## 3. Response Functions of Silicon Photodiode Current Meters

Gain versus frequency curves adequately describe the dynamic characteristics of analog control loops. Most frequently the responsivity (gain) products of the individual components of the open loop are shown on a log amplitude scale versus log frequency. These characteristics can be constructed quickly and fairly accurately by approximating the curves by piecewise linear regions. The construction rules are easy. E.g., a constant in the gain equation gives a horizontal line. 1 + *jωτ* in the denominator gives a roll-off. Roll-off points are also called “poles”. The slope of the roll-off is − 20 dB per decade. 1 + *jωτ* in the numerator, gives a roll-on. A roll-on point is also called “zero”. The slope of the roll-on is − 20 dB per decade. The interconnected straight-line characteristics realized this way are called idealized Bode plots [12].

### 3.1 Voltage Gain Plot of a Si Photodiode Current Meter

The frequency dependent voltage amplification of a photodiode short circuit current meter is described by Eq. (7). The shape of this function will be illustrated first on the widely used silicon photodiode, Hamamatsu Model S1226-8BQ^1^. This photodiode has an active area of 1/3 cm^2^. The photodiode was purchased with a selected *R*_S_ = 6.5 GΩ. The junction capacitance is typically *C*_j_ = 1.3 nF. This photodiode was tested earlier [3] and a noise floor of 0.1 fA was measured with an electrical bandwidth of 1.25 mHz. When the bandwidth was increased to 0.3 Hz, the noise floor increased to 0.6 fA. The feedback resistor was *R* = 10^11^ Ω and the estimated stray capacitance was *C* = 2 pF. The 3 dB open-loop roll-off frequency of the OPA128LM operational amplifier used is *f*_i_ = 3 Hz. This OA was chosen for low input current (40 fA) and a peak-to-peak current noise of 2.3 fA. This selection was necessary to keep the effect of the OA input current small on the very high source resistance (parallel connected *R* and *R*_s_). The dc open-loop gain of the operational amplifier is 110 dB.

**Fig. 2:**
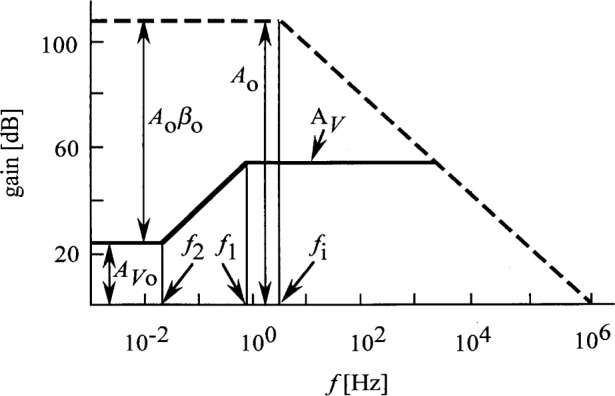
Closed-loop voltage gain (solid lines) of a Hamamatsu S1226-8BQ silicon photodiode current meter. *R*_S_ = 6.5 GΩ, *C*_j_ = 1.3 nF, *R* = 10^11^ Ω, and *C* = 2 pF. The dashed line shows the open loop gain of the OPA 128LM operational amplifier without any feedback.

A time constant of *τ*_1_ = *RC* = 0.2 s gives a signal roll-off frequency of *f*_1_ = 0.8 Hz. From *τ*_2_ = 8 s, the roll-on frequency is *f*_2_ = 0.02 Hz. The roll-off and roll-on slopes are − 20 dB per decade and 20 dB per decade, respectively. *A_V_*_o_ = 16.4 from Eq. (6). In Fig. 2, log *A_V_* versus log frequency is shown, together with the frequency dependent open loop gain of OA. The figure shows that the voltage amplification increases by 1.5 decade when the frequency increases from 0.02 Hz to 0.8 Hz. The photocurrent measuring electrical bandwidth can be limited by a low-pass filter or integrating DVM connected to the output of the photocurrent meter. If this out-of-loop limiting bandwidth is smaller than 0.02 Hz, the noise boosting effect of the photocurrent measuring circuit will be rejected. In this case, the measurement will be slow. However, when the measurement bandwidth is 0.3 Hz, as before [3], or larger, the OA input voltage noise components will be amplified in the 0.02 Hz to 0.8 Hz frequency range.

The shape of the area between the OA open loop gain curve and the *A_V_* voltage amplification curve gives information about the dynamic characteristics of the photocurrent measuring analog control loop. The information about the loop performance can be made more clear and more understandable if this area is illustrated by the Bode plot of the loop gain.

### 3.2 Loop Gain Plot of a Si Photodiode Current Meter

The frequency dependent loop gain of the open analog control loop can be written from Eqs. (5) and (12):

$$G=A_{o}\beta_{o}\frac{1}{1+j\omega \tau_{i}}\frac{1+j\omega RC}{1+j\omega \frac{RR_{s}C+RR_{s}C_{j}}{R+R_{s}}},$$ (17)

where *RC* = *τ*_1_ is the integrating time constant in Eq. (7) (noise voltage gain). Here *τ*_1_ is a differentiating time constant. Similarly, *τ*_2_ of Eq. (8) was a differentiating time constant in the noise voltage gain equation. In Eq. (17), *τ*_2_ is an integrating time constant. The OA time constant, *τ*_i_, is always an integrating type time constant.

**Fig. 3:**
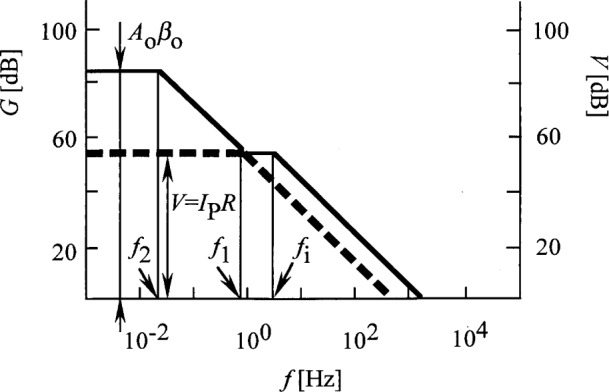
Loop gain plot of the Hamamatsu S1226-8BQ Si photodiode current meter. *R*_S_ = 6.5 GΩ, *C*_j_ = 1.3 nF, *R* = 10^11^ Ω, and *C* = 2 pF. The solid curve shows the gain of the open control loop. The dashed curve shows the current response function as matched at its 3 dB roll-off point to the loop gain function.

Figure 3 shows the Bode plot [12] of the open photocurrent measuring control loop, where *β*_o_ = 0.06, as calculated from Eqs. (4) and (5) with *R* = 10^11^ Ω. *A*_o_ *β*_o_ = 18000. The solid curve shows the dynamic characteristics of the analog control loop. The curve intercepts the frequency axis with a slope of − 20 dB per decade showing that the phase shift (phase lag) in the open loop at this frequency is not more than − 90°. This phase shift corresponds to one integrating time constant. This − 90° phase shift, together with the − 180° phase shift of the negative feedback, results in a maximum phase shift of − 270°. Since this phase shift is less than − 360°, oscillations will not occur. Also, the loop gain, *G* = *Aβ*, is equal to or larger than 1000 (60 dB) from 0 Hz to about the 3 dB roll-off frequency of the signal response curve. The large *G* in the low frequency interval gives a high current-to-voltage conversion accuracy. The dashed photocurrent-to-voltage response curve, which is described by Eq. (15), was matched at its 3 dB point to the loop gain function.

**Fig. 4:**
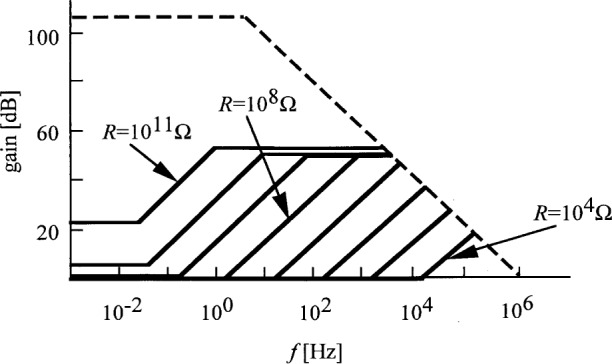
Closed-loop voltage gain characteristics of the Hamamatsu S1226-8BQ Si photodiode current meter when the feedback resistors are changed from 10^11^ Ω to 10^4^ Ω. *R*_S_ = 6.5 GΩ, *C*_j_ = 1.3 nF, and *C* = 2 pF. The dashed line shows the open loop gain of the OPA 128LM operational amplifier, without any feedback.

The overall electrical bandwidth of photocurrent measurements greatly depends on *R*. This is true for both the signal response in Eq. (15) and the loop bandwidth described by Eq. (17). The speed of the above analyzed high sensitivity photocurrent meter is slow because of the very large *R*. The speed can be increased by decreasing *R*. However, smaller *R* decreases the photocurrent responsivity. Figure 4 shows the voltage gain curves of the Hamamatsu S1226-8BQ silicon photodiode current meter. The noise boosting effect does not disappear with decreasing *R*. Decreasing *R* shifts the noise boosting interval to higher frequencies. Small feedback resistors are used when the photocurrent is large. In these cases, the noise boosting effect is less of a problem.

**Fig. 5:**
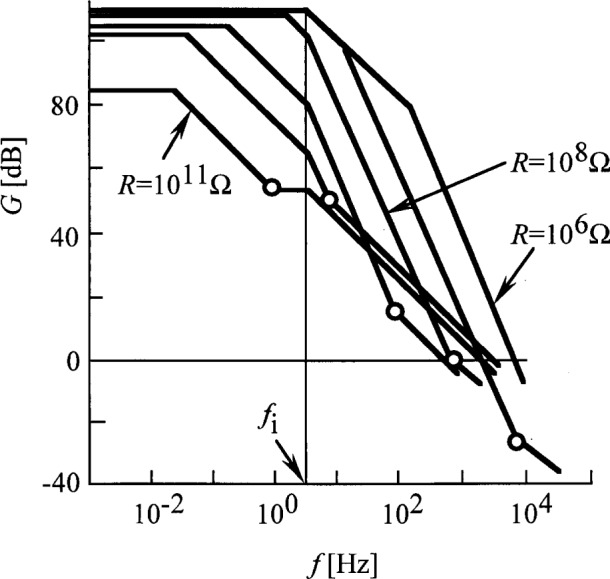
Loop gain characteristics of the Hamamatsu S1226-8BQ photodiode current meter when the feedback resistors are changed from 10^11^ Ω to 10^6^ Ω. *R*_S_ = 6.5 GΩ, *C*_j_ = 1.3 nF, and *C* = 2 pF (stray). No external capacitors are connected parallel to the feedback resistors. The open circles show the 3 dB roll-off points of the photocurrent response. They are matched to the loop gain curves for each *R*.

Decreasing *R* will increase both the loop bandwidth and the low frequency loop gain. Figure 5 shows the different loop gain plots of the Hamamatsu S1226-8BQ silicon photodiode circuit when *R* changes from 10^11^ Ω to 10^6^ Ω. Similarly to Fig. 3, *f*_2_ < *f*_1_ for all feedback resistors when *C* = 2 pF = constant. When *R* decreases, both the roll-off frequency, *f*_2_, and the roll-on frequency, *f*_1_, increase. With decreasing *R*, *f*_1_, which is equal to the signal 3 dB roll-off point, shifts towards the frequency axis. At *R* = 10^8^ Ω, *f*_1_ reaches the log *f* axis. At this frequency, the phase lag in the open loop is − 135°. *τ*_i_ and *τ*_2_ integrating time constants shift − 180°, and *τ*_1_ differentiating time constant gives a phase lead of + 45°. If *R* is further decreased, the phase shift at high frequencies (close to the unity gain cut-off frequency) can reach − 180°, resulting in oscillations in the closed loop. In order to increase stability and accuracy, *f*_1_ has to be decreased by increasing *τ*_1_. In our previous experimental circuits [1,2,3], when *R* was 10^6^ Ω or smaller, *τ*_1_ was increased using external capacitors, parallel connected to the feedback resistors. Changing the feedback impedance by tuning the external parallel capacitor [15] can fundamentally modify the frequency dependent characteristics of the photocurrent meter.

### 3.3 Frequency Compensation of Current Meters

**Fig. 6:**
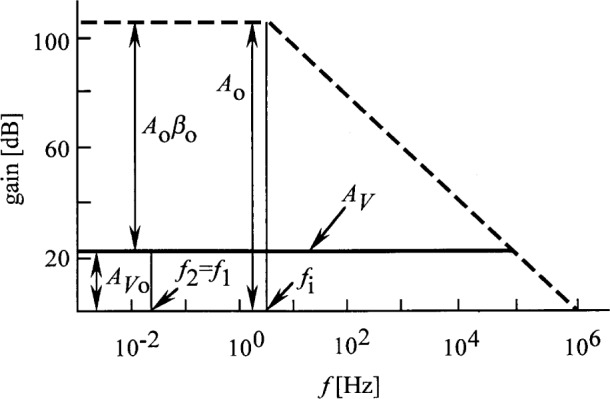
Frequency compensated closed-loop voltage gain of the Hamamatsu S1226-8BQ Si photodiode current meter. *R*_S_ = 6.5 GΩ, *C*_j_ = 1.3 nF, *R* = 10^11^ Ω, and *C* = 80 pF. The dashed line shows the open loop gain of the OPA 128LM operational amplifier, without any feedback.

The noise boosting effect can be eliminated if *τ*_1_ = *τ*_2_. This compensation can be done easily by connecting an external capacitor of 78 pF parallel to *R* = 10^11^ Ω. The sum of the 78 pF and the 2 pF stray capacitance will give the necessary *C* = 80 pF to achieve the frequency compensation. In this case, *τ*1 will be 8 s. After the compensation, the shape of the solid curve, *A_V_*, in Fig. 2 will change to a straight line, as shown in Fig. 6. The improved noise amplification will be *A_V_* = *A_V_*_o_ for all frequencies within the loop.

**Fig. 7:**
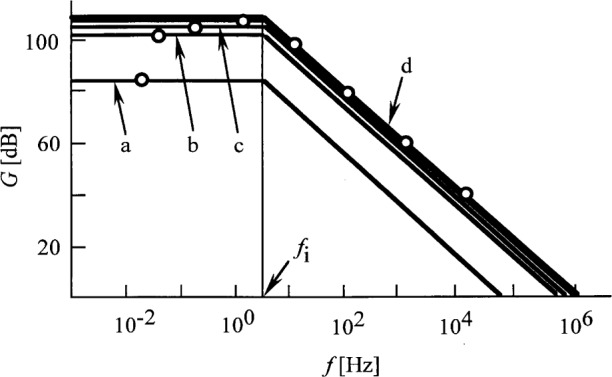
Compensated loop gain characteristics of the Hamamatsu S1226-8BQ Si photocurrent meter. *R*_S_ = 6.5 GΩ and *C*_j_ = 1.3 nF. The feedback impedances are changed: a) *R* = 10^11^ Ω, *C* = 80 pF; b) *R* = 10^10^ Ω, *C* = 510 pF; c) *R* = 10^9^ Ω, *C* = 1.1 nF; d) *R* = 10^8^ Ω to 10^4^ Ω and *C* = 1.3 nF. The open circles show the 3 dB roll-off points of the photocurrent response functions. They are matched to the loop gain curves for all feedback resistors, after compensation.

If the compensation is made for all feedback resistors, the shape of the loop gain curves of Fig. 5 will be different. In each compensation, *τ*_1_ canceled *τ*_2_; therefore, *τ*_i_ became the only integrating time constant in the loop. Figure 7 shows the compensated loop gain curves for different feedback resistors. The loop bandwidth increased significantly because of the compensation. Without frequency compensation the loop gains were high enough only for dc and low signal frequencies. For feedback resistors between 10^10^ Ω and 10^4^ Ω, the loop dynamic characteristics are very similar. There are no oscillation problems because the phase shift is less than − 90° even at high loop frequencies. The 3 dB roll-off points of the photocurrent response function of Eq. (15) are also shown for the different feedback impedances. For all feedback resistors, the compensated signal 3 dB points limit the speed of the photocurrent meter. The loop gains at the signal 3 dB points are always larger than 100. If a minimum loop gain of 1000 is required to achieve a 0.1 % relative standard uncertainty in photocurrent measurements, the signal frequency at the lowest signal gain of *R* = 10^4^ Ω has to be limited to about 1 kHz. In the case of a wide dynamic signal range, this frequency limitation is not a problem, because the signal frequency limit is much lower at high signal gains. The very low input current OPA128LM operational amplifier seemed to be the best selection for this very high sensitivity but very slow photocurrent meter.

**Fig. 8:**
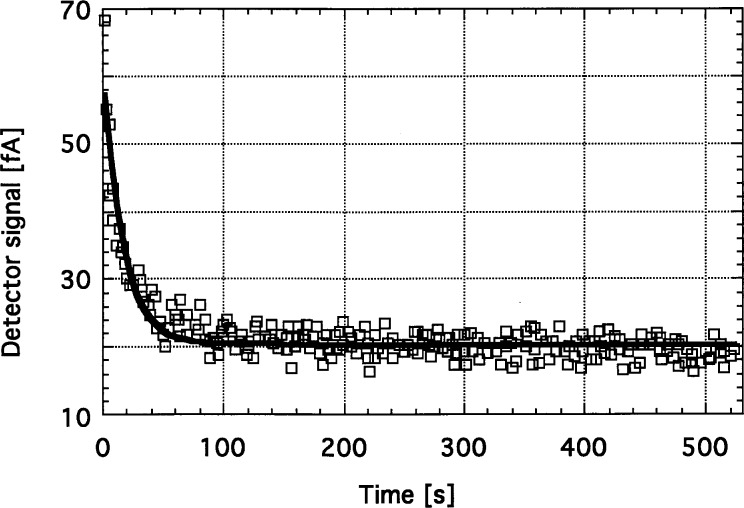
The measured settling time of the Hamamatsu S1226-8BQ silicon photocurrent meter when *R* = 10^11^ Ω. The time constant from the fit is 16.2 s.

The frequency compensation made the signal response of the current meter slower. This can be a problem when *R* is high. E.g., for *R* = 10^11^ Ω, the *τ*_1_ = 8 s integrating time constant of the signal response requires about a 1 min wait for the digital voltmeter (DVM) to measure the signal accurately at the output of the current meter. The long waiting time before each DVM measurement is also necessary because of the roughly 2 min settling time of the OPA128LM operational amplifier when operated with this large feedback resistor. The measured settling time of the above discussed silicon photodiode current meter is shown in Fig. 8. The figure shows the output signal change of the meter after the shutter is closed. The duration of one measured point on the figure was determined by the integration time of the DVM, which was equal to the time of one power line cycle. This corresponds to an electrical bandwidth of 30 Hz [3]. A 16.2 s time constant was obtained from the curve fit to the measured data when an *R* = 10^11^ Ω feedback resistor was used. No external feedback capacitor was applied in this measurement. When *R* = 10^10^ Ω was selected, the settling time constant became shorter than the duration of one power line cycle.

As shown in Fig. 4, the dc voltage noise amplification decreased almost a decade when *R* was reduced from 10^11^ Ω to 10^10^ Ω. At the same time, the photocurrent response also decreased by a factor of 10. Decreasing *R* results in a smaller source resistance noise for the input of the OA. As a result of the similar signal and noise changes, the signal-to-noise ratio for the output of the current meter is similar for these two signal gain selections.

Based on the above settling time and signal to noise ratio measurements, we conclude that *R* = 10^10^ Ω produces a faster measurement than *R* = 10^11^ Ω with similar photocurrent limit sensitivity.

### 3.4 Chopped Radiation Measurement

When dc or low frequency operation in a photocurrent measuring circuit is too slow to satisfy a certain measurement speed requirement, different photodiode and operational amplifier selections are needed.

**Fig. 9:**
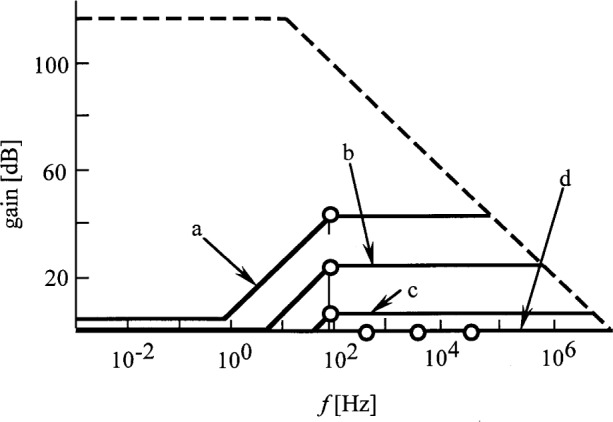
Closed-loop voltage gains (solid curves) of a Hamamatsu S5226-8BQ Si photodiode ac current meter. *R*_S_ = 1 GΩ and *C*_j_ = 430 pF. The dashed curve shows the open loop gain of the OPA627BM operational amplifier, without any feedback. Partial frequency compensations were made at 80 Hz: a) *R* = 10^9^ Ω and *C* = 2 pF; b) *R* = 10^8^ Ω and *C* = 20 pF; and c) *R* = 10^7^ Ω and *C* = 200 pF. Full compensations are shown by d) where *R* = 10^6^ Ω to 10^4^ Ω and *C* = 432 pF. The 3 dB roll-off points of the signal response curves are matched to the voltage gain curves and are shown with open circles.

The frequency dependent photocurrent-to-voltage conversion is described by Eq. (15). The *RC* time constant of the feedback impedance determines the bandwidth of the signal measurement. This time constant has to be small enough to keep the 3 dB signal roll-off frequency a decade higher than the frequency of the signal to be measured. E.g., with *C* = 2 pF, *R* can not be larger than 10^9^ Ω to use a chopping frequency of 8 Hz. In this case *τ*_1_ = 2 ms and *f*_1_ = 80 Hz. Because the voltage amplification can not be smaller than unity, for *R* < 10^9^ Ω the photodiode shunt resistance should not be larger than 10^9^ Ω. Also, if the junction capacitance is low enough, the *f*_2_ roll-on frequency of the voltage gain curve of Eq. (7) can be selected higher than the signal (chopping) frequency. A possible detector choice is the Hamamatsu S5226-8BQ silicon photodiode. This device has an active area of 1/3 cm^2^. The shunt resistance is 1 GΩ and the junction capacitance is 430 pF. Figure 9 shows the voltage gain curves for the Hamamatsu S5226-8BQ silicon photodiode when used with the OPA627BM low noise and wide band operational amplifier. Partial frequency compensations were performed for all of those *R* where *f*_2_ < 80 Hz. In these cases, the signal 3 dB points were tuned to 80 Hz. For those *R* where *f*_2_ > 80 Hz, full frequency compensations were obtained. Frequencies *f*_1_ were decreased to be equal to *f*_2_. For each fully compensated gain (*R* = 10^6^ Ω, 10^5^ Ω, and 10^4^ Ω), the sum of the stray and external capacitances was 432 pF. The signal 3 dB roll-off points are matched to the voltage gain functions for each *R* and are shown with open circles. The noise boosting effect disappeared after full compensations and decreased for partial compensations. If a chopping frequency of 8 Hz is selected and *R* = 10^8^ Ω is used as a maximum feedback resistor, the noise amplification will be practically unity.

The OA selection criteria for rapidly changing optical radiation is different than the earlier discussed very slow signal measurements. In the presently discussed ac measurement, the source resistance (parallel connection of *R* and *R*_s_) was selected to be smaller than in the previously discussed dc and low frequency measurements. Consequently, the input bias current of the OA does not have to be extremely low. However, low noise, fast settling time, and fast operation are important OA requirements. The OPA627BM dielectrically isolated OA satisfies these expectations. This operational amplifier has low-noise, equal to bipolar-input amplifiers, larger bandwidth than that of FET input operational amplifiers, and the minimum slew rate is 40 V/μs.

**Fig. 10:**
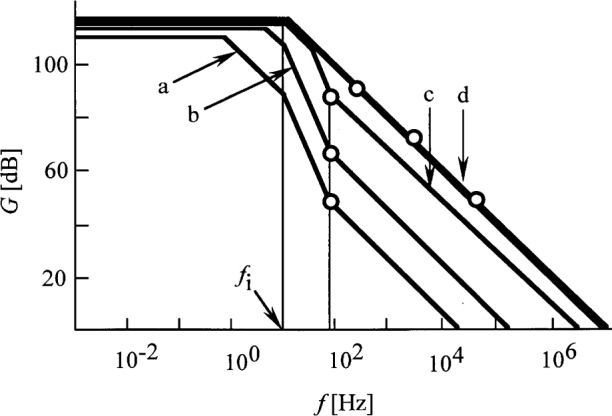
Loop gain curves of an ac photocurrent meter using a Hama matsu S5226-8BQ Si photodiode. *R*_S_ = 1 GΩ and *C*_j_ = 430 pF. OPA627BM operational amplifier is used. Partial frequency compensations are made at 80 Hz: a) *R* = 10^9^ Ω and *C* = 2 pF; b) *R* = 10^8^ Ω and *C* = 20 pF; and c) *R* = 10^7^ Ω and *C* = 200 pF. Full compensations are shown by d) where *R* = 10^6^ Ω to 10^4^ Ω and *C* = 432 pF. The signal 3 dB roll-off points are matched to the loop gain curves and are shown with open circles.

The loop gain characteristics of the optimized ac silicon photodiode current meter for the different feedback impedances are shown in Fig. 10. The signal 3 dB roll-off points are matched to the loop gain curves and are shown again with open circles. The loop gain is higher than 300 at each signal 3 dB roll-off points for all *R* selections. Because of the large loop gains at signal frequencies smaller than the 3 dB point, the analog control loop errors are small and the implementation of Eq. (16) is accurate. E.g., *G* > 10^3^ for signal frequencies smaller than 40 Hz.

If the radiation is chopped, a lock-in amplifier is usually connected to the output of the current meter. The lock-in, which is synchronized with the radiation chopper, performs a phase sensitive rectification of its input signal. The low-pass filter, coupled to the output of the lock-in, smooths the signal. The filter should be properly designed to take fast enough readings when the optical radiation changes. Usually, active filters (e.g., Bessel) are used to optimize filter characteristics [9]. If a very small bandwidth is realized by the low-pass filter (for an improved signal to noise ratio), the measurement will be very slow.

## 4. Conclusions

In addition to signal range and sensitivity, speed can be an important issue in photodiode short circuit current measurements. In order to calculate the frequency dependent signal and noise gains of different photodiode current meters, a detailed analysis of the photocurrent measuring analog control loops has been described. First, the most important gain equations were determined and then the current-to-voltage gain *A_I_*, voltage gain *A_V_*, and loop gain *G* were optimized for the signal frequencies. Both the active and passive components of photodiode current measuring circuits can be determined using the described method. Photodiodes can be selected for shunt resistance and junction capacitance according to the sensitivity and speed requirements of a measurement. The feedback impedances for the selected operational amplifier can be matched to the impedance of the selected photodiode. As a result of component selections and frequency compensations, the signal-to-noise ratios can be optimized for the outputs of the photocurrent meters, and improved loop gains can be achieved for reasonably wide frequency ranges. Because of improved loop gain and bandwidth, the accuracy of the photocurrent-to-voltage conversion is increased for higher signal frequencies. As a result of photodiode circuit optimization, the signal roll-off of large area silicon photodiode light meters can be increased to 80 Hz even at a signal gain of 10^9^ V/A.

## Footnotes

1 Certain commercial equipment, instruments, or materials are identified in this paper to foster understanding. Such identification does not imply recommendation or endorsement by the National Institute of Standards and Technology, nor does it imply that the materials or equipment identified are necessarily the best available for the purpose.
